# A review of probiotics studies in HIV research suggests improved immunological presentation and preservation of viral host restrictive factors of TH17 in HIV patients

**DOI:** 10.1186/1742-4690-9-S1-P22

**Published:** 2012-05-25

**Authors:** M Selbovitz, Keller, Miller, Moore, Farmer, Bray

**Affiliations:** 1 Health Action, Bronx, USA

## Background

Recovery of gut mucosal immune system is slow and incomplete during HAART therapy, leading to elevated inflammation rates, increased mitochondrial damage and the pathogenesis of replicatively competent escape mutations. A recent study of Ganeden BC30, Bacillus coagulans GBI-30, demonstrated safety, increased CD4+ counts in patients on HARRT, and a significant increase in CD69 and maturation of dendritic cells in vitro. Lactobacillus rhamnosus GR-1 and Lactobacillus reuteri RC-14 demonstrate efficacy in treating naïve HIV patients against gastrointestinal and urogenital infections . Restoration of viral host restrictive factors of Th17 cell lines in GALT may prevent the evolving viral diversity, provoking increased CD4 presentation and regulation of inflammatory cytokine levels responsible for increased viral replication.

## Methods

Metagenomic sequencing analysis has shown alternations in intestinal microbiota in HIV patients. Measurements of immunological parameters including Serum cytokine levels and total serum IgE levels (CD4 lymphocyte count, CBC, levels of TNFÎ±, NFAT, IL-12, IL-10, and G-CSF [Kim, et.al. 2006]) were measured in studies reviewed here.

## Results

A recent study of Ganeden BC30, bacillus coagulans GBI-30, 6086 demonstrated safety, and an increase in %CD4, a significant increase in CD69 activation and maturation of dendritic cells in HIV patients on HARRT and improvement in GI function. Lactobacillus rhamnosus GR-1 and Lactobacillus reuteri RC-14 have demonstrated efficacy treating naïve HIV patients gastrointestinal and urogenital infections and prevent diarrhea and increased CD4 T-lymphocyte percentages in HIV patients.

## Conclusions

Intestinal microbiota are integral to the homeostasis and functioning of immune cells. The loss of intestinal flora by HIV infection is severely detrimental to the recombination of CD4 cells. Restoring proper biodiversity in the gut by safe, efficacious probiotics demonstrates promise in increasing Th17 cells by restoring GALT. GanedenBC30 may down regulate TNF-a and other inflammatory cytokines and mitigate ARV-related SAEs.

Data suggests that replenishment of Th17 CD4 cells in the gut mucosa during HAART correlates with improved function of the gut mucosal immune system and its function.. Studies by NIH of probiotics to lower microbial translocation and immune activation in HIV-infected adolescents.

